# Efficient *in vitro *RNA interference and immunofluorescence-based phenotype analysis in a human parasitic nematode, *Brugia malayi*

**DOI:** 10.1186/1756-3305-5-16

**Published:** 2012-01-13

**Authors:** Frédéric Landmann, Jeremy M Foster, Barton E Slatko, William Sullivan

**Affiliations:** 1 Department of Molecular, Cell and Developmental Biology, University of California Santa Cruz, 1156 High Street, Santa Cruz, CA 95604, USA; 2 Division of Molecular Parasitology, New England Biolabs, Inc., 240 County Road, Ipswich, MA 01938, USA

**Keywords:** RNAi, nematode, immunostaining, *Brugia*, filaria

## Abstract

**Background:**

RNA interference (RNAi) is an efficient reverse genetics technique for investigating gene function in eukaryotes. The method has been widely used in model organisms, such as the free-living nematode *Caenorhabditis elegans*, where it has been deployed in genome-wide high throughput screens to identify genes involved in many cellular and developmental processes. However, RNAi techniques have not translated efficiently to animal parasitic nematodes that afflict humans, livestock and companion animals across the globe, creating a dependency on data tentatively inferred from *C. elegans*.

**Results:**

We report improved and effective *in vitro *RNAi procedures we have developed using heterogeneous short interfering RNA (hsiRNA) mixtures that when coupled with optimized immunostaining techniques yield detailed analysis of cytological defects in the human parasitic nematode, *Brugia malayi*. The cellular disorganization observed in *B. malayi *embryos following RNAi targeting the genes encoding γ-tubulin, and the polarity determinant protein, PAR-1, faithfully phenocopy the known defects associated with gene silencing of their *C. elegans *orthologs. Targeting the *B. malayi *cell junction protein, AJM-1 gave a similar but more severe phenotype than that observed in *C. elegans*. Cellular phenotypes induced by our *in vitro *RNAi procedure can be observed by immunofluorescence in as little as one week.

**Conclusions:**

We observed cytological defects following RNAi targeting all seven *B. malayi *transcripts tested and the phenotypes mirror those documented for orthologous genes in the model organism *C. elegans*. This highlights the reliability, effectiveness and specificity of our RNAi and immunostaining procedures. We anticipate that these techniques will be widely applicable to other important animal parasitic nematodes, which have hitherto been mostly refractory to such genetic analysis.

## Background

Filarial nematodes cause debilitating pathologies in tropical areas with > 1 billion people at risk. Current anthelmintic drugs predominantly target larval stages only, and a developing resistance has been indicated [1-3]. Since almost all filarial species that cause disease in humans rely on the bacterial endosymbiont *Wolbachia *for proper embryogenesis, development and viability, these symbionts have become a major drug target for novel anti-filarial strategies [4,5]. It is crucial to characterize the molecular and cellular mechanisms underlying *Wolbachia *transmission, since their segregation patterns in the embryo determine the localization in adult hypodermal chords [6]. Such knowledge could lead to the molecular identification of new drug targets.

Despite the published genomes of *Brugia malayi *[7], a causative agent of lymphatic filariasis and elephantiasis, and its *Wolbachia *endosymbiont [8], both the developmental biology of filarial nematodes and the mutualism with *Wolbachia *are still poorly understood. The free living nematode *Caenorhabditis elegans*, remains the closest animal model to date. While *C. elegans *represents a valid model for general aspects of nematode biology [9], its estimated > 500 million year separation from *B. malayi *[10] and free-living lifestyle leave questions related to parasitism unanswered [11]. Furthermore, *C. elegans *data cannot inform on mutualism with *Wolbachia *since within the Nematoda this bacterium appears limited to parasitic nematodes within the family Onchocercidae [4]. Therefore inference of gene function or biological processes in parasitic species based on data from *C. elegans *should be made with caution. While reverse genetic tools such as RNA interference (RNAi) are routinely used in *C. elegans *research to characterize gene function [12], RNAi experiments in animal parasitic nematodes have proven notoriously challenging [13-18] with few successes reported. A bioinformatic study comparing the RNAi effector protein complements of various animal parasitic nematodes to that of *C. elegans *found that while quantitative differences exist (with *C. elegans *having the richest complement), all species were qualitatively similar and should be RNAi-competent [19]. Indeed, there are a few reports of successful RNAi in filarial nematodes [20-26] although several of these studies targeted the same genes. Various explanations for the limited success of RNAi in animal parasitic nematodes have been proposed [15,19,27].

We describe an efficient *in vitro *RNAi procedure we have developed to successfully copy RNAi-induced phenotypes observed in *C. elegans *during early embryogenesis in the parasitic nematode *B. malayi*. We also further optimized our immunofluorescence protocols [6] to permit detailed characterization of the embryonic RNAi phenotypes. These methodologies may be extended to genes expressed in the germ line or in adult tissues. Our results demonstrate that RNAi can be a reliable and effective tool for gene function studies in parasitic species. The enhanced procedures for both RNA delivery and subsequent immunofluorescence-based phenotype analysis open the way to address fundamental questions in parasitic nematode biology, emancipation from the *C. elegans *model, and investigation of the interaction between the filarial parasite and its *Wolbachia *symbiont.

## Methods

See Additional file 1 for a detailed step-by-step protocol and Additional files 2, 3, 4 for illustration of key steps in the procedure.

### Preparation of heterogeneous short interfering RNA (hsiRNA)

Total RNA was prepared from adult *B. malayi *(TRS Labs, Athens, GA, USA) by established methodology http://www.filariasiscenter.org/molecular-resources/protocols and ~ 700 ng RNA used as template for production of cDNA using the ProtoScript M-MuLV First Strand cDNA Synthesis Kit according to the manufacturer's instructions (New England Biolabs. Ipswich, MA, USA).

DNA templates for *in vitro *transcription were generated by PCR using Crimson Taq DNA Polymerase (New England Biolabs). PCR primers contained T7 promoter sequence followed by two guanine bases at their 5' ends for transcription by T7 RNA polymerase and enhanced transcription yield. Primers were designed to yield a PCR product corresponding to ~500 bp of the transcript selected for gene silencing. Typically a region towards the 5' end of the open reading frame was selected and primers that would span an intron in genomic DNA (gDNA) were designed. This allows differentiation between amplification from 1^st ^strand cDNA and any residual gDNA present in the cDNA preparation. However, we also demonstrated that RNA transcribed from gDNA that included introns was effective in gene silencing (see *par-1 *example). Primers having potential to amplify other *B. malayi *genes or to generate amplicons with considerable sequence similarity to non-target transcripts were avoided. PCR mixes (50 μl) generally contained ~100 ng template cDNA, 0.4 μM each primer, 0.2 mM dNTPs and 0.25 μl (1.25 U) polymerase in 1 × buffer. Between 1 and 5 μl of the PCR product was loaded on to a 1.5% agarose gel to assess the quality and size of the amplicon.

dsRNA corresponding to the gene to be targeted by RNAi was prepared using the HiScribe T7 In Vitro Transcription Kit according to the recommended protocol (New England Biolabs). Approximately 1 μg of PCR product was used in a 80 μl reaction without any purification prior to transcription. The reaction was incubated at 42°C for 2.5 hours then checked by loading 1 μL on a 1.5% agarose gel alongside dsRNA Ladder-A (New England Biolabs). When suitable RNA products were visualized, the remainder of the sample was purified by isopropanol precipitation and the RNA pellet resuspended in 50 μL RNase-free water.

The long dsRNA was processed to heterogeneous short interfering RNA (hsiRNA) using ShortCut RNase III as recommended (New England Biolabs). The entire preparation of dsRNA (50 μl) was used in a 100 μl reaction. The hsiRNA was purified by ethanol precipitation, resuspended in 60 μL RNase-free water, and an aliquot examined by gel electrophoresis (2% agarose) alongside siRNA Marker (New England Biolabs). Quantification of hsiRNA was achieved using a Nanodrop or standard spectrophotometer.

### In vitro hsiRNAi by soaking

In a laminar flow hood, the appropriate amount of hsiRNA was placed in wells of a 12-well cell culture plate to which 1 ml Worm Culture Medium (WCM) was added. [WCM: RPMI-1640 cell culture medium with L-glutamine (Invitrogen, Carlsbad, CA, USA), 1% glucose, 100 × antibiotic-antimycotic solution (A-5955; Sigma-Aldrich, St Louis, MO, USA), 10% fetal bovine serum inactivated at 56°C for 30 minutes (Invitrogen)]. Two female worms were transferred to each well using a curved pick (see Additional file 2). The medium was replaced every 12 hours by pipetting hsiRNA into new wells, adding fresh WCM and then transferring the worms (see Additional file 3). Typically 1 μM hsiRNA was used but the concentration was increased up to 5 μM in instances where no effect was observed. Controls included worms cultured similarly but in the absence of any RNA and worms cultured in an equivalent concentration of Lit28i polylinker ShortCut siRNA mix (New England Biolabs).

Worms were incubated in a 37°C, 5% CO_2 _incubator. Exposure to the hsiRNA mixture was continued for between 2 and 5 days.

### Embryo and Tissue Collection and Fixation

We optimized and simplified our previously published protocol [6]. The worms from each well were transferred to a drop of 10 μl 1 × PBS on a microscope slide using the curved pick. For maximizing recovery of embryos, the worms were diced into small fragments using a razor blade (see Additional file 4). To obtain larger anatomical structures, such as the ovaries, fewer cuts were made under a dissection microscope, close to the posterior tip for instance, since the hydrostatic pressure expels organs out of the pseudocoelom and into the PBS. Once the ovaries, uteri, or intestine were expelled, they were cut into shorter fragments to facilitate subsequent tissue penetration by antibodies. The fragments and embryos were transferred to a microcentrifuge tube by pipetting 180 μL 1 × PBS, 1% NP-40 across the slide surface and into the tube (see Additional file 4). Next, 20 μl 32% paraformaldehyde and 2 volumes heptane (~400 μl) were added. The tube was vortexed for one minute, then left on a rotator for 20 minutes. The contents were pelleted in a microcentrifuge (2000 × *g*, 1 min), the supernatant removed and the pellet resuspended in 1 ml PBST-BSA (1 × PBS, 0.02% Triton X-100, 2% BSA (Fraction V; Fisher Scientific, Pittsburgh PA, USA)) by rotation for 5 minutes.

For embryo staining specifically, the tube was centrifuged again and the pellet transferred to a microscope slide using a Pasteur pipette. A coverslip was applied and pressed down gently using a paper towel to absorb excess PBST. Using forceps, the slide was held submerged in liquid nitrogen until the liquid stopped boiling. The slide was placed on the bench and the coverslip removed with a razor blade. Once the embryos had thawed they were washed into a microcentrifuge tube by pipetting PBST-BSA over the slide surface (see Additional file 4). This "freeze-crack" step removes a high proportion of the eggshells, thereby increasing the yield of high quality immunostaining.

These protocols were optimized for young embryos and tissue fragments, and permit immunostaining of all embryos up to about the 20 cell stage. However, the proportion of stained embryos decreases with increasing development stage, possibly because of changes in the eggshell composition, that increase resistance to the treatment.

### Alternative treatment for fixation of older embryos

To increase the staining of older embryos (i.e. after morphogenesis begins), the following step was used instead of the "freeze-crack" procedure. After dicing the worms, the tissues/embryos were transferred from the slide to a microcentrifuge tube using 180 μl 1 × PBS, 20% bleach (see Additional file 4) then vortexed for no more than 30 seconds. PBS (1 ml) was added, the tube shaken then centrifuged immediately (2000 × *g*, 1 min). The supernatant was removed with a pipette then 180 μl 1 × PBS, 1% NP-40 was added to the pellet followed by 20 μl 32% paraformaldehyde and 2 volumes heptane (~400 μl). The sample was then processed as described above resulting in embryos in PBST-BSA.

### RNase treatment (optional)

When DNA was to be stained with propidium iodide the samples were treated with RNase prior to antibody incubations. The tubes containing samples in PBST were centrifuged (2000 × *g*, 1 min), the supernatant removed, and replaced by 1 ml 10 mg/ml RNaseA (Sigma Aldrich, Catalog No. R4875) in PBST. The sample was rotated overnight at 4°C.

[For subsequent analysis using a fluorescence microscope or a confocal equipped with a UV laser, DNA can alternatively be stained at the end of the procedures with DAPI-containing mounting medium.]

### Staining and Immunostaining

Samples were spun (2000 × *g*, 1 min) to remove PBST (or PBST-RNase), and resuspended in 500 μl fresh PBST. Primary antibodies were diluted into the sample, which was then rotated overnight at 4°C. Next day, the samples were centrifuged (2000 × *g*, 1 min), the supernatant removed and replaced by 1.5 ml PBST and the tube rotated at room temperature for > 15 minutes. This step was repeated twice but the sample resuspended in 500 μl PBST after the final wash. Secondary fluorochrome-conjugated antibodies were added according to the manufacturer's recommended dilution and the samples rotated overnight at 4°C. The tubes were centrifuged (2000 × *g*, 1 min), the supernatant discarded and 1.5 ml PBST added. The tubes were rotated for > 15 minutes then centrifuged again. The supernatant was discarded, 1.5 ml PBST added and, if required, 20 μl of 1 mg/ml propidium iodide (Invitrogen) added. The tube contents were mixed by shaking for 20 seconds, then centrifuged again. The supernatant was removed and replaced by 1.5 ml PBST. The tube was again shaken for 20 seconds, centrifuged, and as much liquid as possible carefully removed using a pipette. About 30 μl mounting medium (e.g. Vectashield, Vector Laboratories, Burlingame, CA, USA) was added to the tube. The mounting medium can contain DAPI if the DNA was not already stained with propidium iodide. The mounting medium and sample were mixed by gently pipetting up and down with a Pasteur pipette then transferred to a microscope slide. At this point, larger adult worm fragments can be discarded or transferred to a second slide using fine tweezers. This reduces the volume between the slide and coverslip, thereby minimizing floatation of embryos during observation. Adult fragments were mounted on a second slide using 30 μl (or more) of mounting medium. Coverslips were added and for embryo preparations, downwards pressure was applied using a paper towel to absorb excess mounting medium and stabilize the embryos. Slides were sealed with transparent nail polish and stored at 4°C in the dark. When DAPI was used in the mounting medium, the slides were stored for > 24 hr prior to microscopy to allow the stain to penetrate embryos and tissues completely.

### Microscopy

Confocal microscope images were captured on an inverted photoscope (DMIRB; Leica Microsystems, Wetzlar, Germany) equipped with a laser confocal imaging system (TCS SP2; Leica) using an HCX PL APO 1.4 NA 63 oil objective (Leica) at room temperature.

Microtubule stainings were performed using the monoclonal DM1α antibody raised against α-tubulin (Cell Signaling Technology, Danvers, MA, USA) at a dilution of 1:100. A Cy5 goat anti-mouse secondary antibody (Invitrogen) was used at 1:250. Actin stainings were performed using the fluorescent Atto 488 phalloidin (Sigma) at a dilution of 1:100.

## Results and discussion

To test RNAi in *B. malayi*, we decided to target orthologs of a number of genes known to be involved in *C. elegans *development and which show clear cellular phenotypes when depleted in that species. We specifically targeted proteins with a structural role, and their regulators.

**Figure 1:**
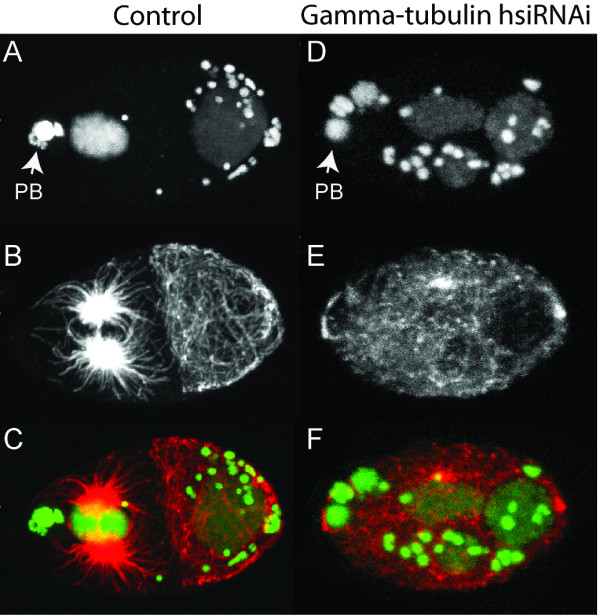
**RNAi against γ-tubulin in *B. malayi *early embryos**. Stained embryos from untreated (A, B, C) or γ-tubulin hsiRNA-treated (D, E, F) worms are shown. In the untreated 2-cell stage embryo (A, B, C), the anterior blastomere (oriented to the left) divides first as evidenced by the star-like spindles (B, C). In contrast, all zygotes from γ-tubulin-treated hsiRNA-treated worms (D, E, F) showed cytokinesis defects (cytokinesis did not occur) leading to polynucleated 1-cell embryos. Propidium iodide reveals the nematode DNA and the *Wolbachia *DNA as large and small dots, respectively (A, D). Immunostaining with α-tubulin antibody (B, E). Merge of DNA (green) with α-tubulin (red) (C, F). Anterior to the left, as indicated by the polar bodies (PB). A 445 bp fragment of the *B. malayi *γ-tubulin cDNA was amplified with primers: γTubF- 5'-TAATACGACTCACTATAGGGCTTCGTGAAGGTGATGCTAC-3' and γTubR 5'-TAATACGACTCACTATAGGGCACGTTCAACAGCATCACTC-3' where the T7 promoter sequence is underlined and followed by a GG nucleotide (see Methods). An annealing temperature of 56°C was used in the PCR. The cDNA fragment was transcribed and RNA diced to hsiRNA as described. Adult females were soaked for 2 days in 100 nM γ-tubulin hsiRNA and tissues fixed on the third day. Control females were kept in the same conditions and included worms cultured without hsiRNA or with a comparable concentration of Lit28i polylinker ShortCut siRNA mix.

**Figure 2:**
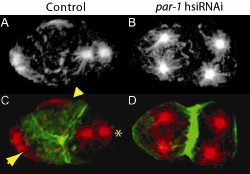
**RNAi against *par-1 *in *B. malayi *early embryos**. Stained embryos from untreated (A, C) or *par-1 *hsiRNA-treated (B, D) worms are shown. In embryos from untreated control worms (A, C) divisions at the 2-cell stage are asynchronous and the anterior blastomere divides first in a transverse orientation giving rise to anterior blastomere a (ABa; tapered arrowhead) and anterior blastomere b (ABb; compact arrowhead). The spindle (star-like structure) in the posterior blastomere (P1; asterisk) rotates to align along the anterior-posterior axis. In contrast, in embryos from *par-1 *hsiRNA-treated worms (B, D) divisions at the 2-cell stage become synchronous as evidenced by the star-like spindles, and the posterior spindle in P1 fails to rotate, remaining transverse as in the anterior blastomere. Immunostaining with α-tubulin antibody (A, B). Merge of α-tubulin (red) with actin (green) as revealed by phalloidin staining (C, D). Anterior to the left. A ~2 kb fragment of the *B. malayi par-1 *locus (corresponding to ~500 bp cDNA) was amplified with primers: par-1F 5'-TAATACGACTCACTATAGG GGAGAGGAATCTTGCCAACGG-3' and par-1R 5'-TAATACGACTCACTATAGG GAACTGCTTGTGCAGATGCGC-3' where the T7 promoter sequence is underlined and followed by a GG nucleotide (see Methods). An annealing temperature of 59°C was used in the PCR. The PCR fragment amplified from gDNA was transcribed and RNA diced to hsiRNA as described. Adult females were soaked for 2 days in 1 μM *par-1 *hsiRNA and tissues fixed on the third day. Control females were kept in the same conditions and included worms cultured without hsiRNA or with a comparable concentration of Lit28i polylinker ShortCut siRNA mix.

**Figure 3:**
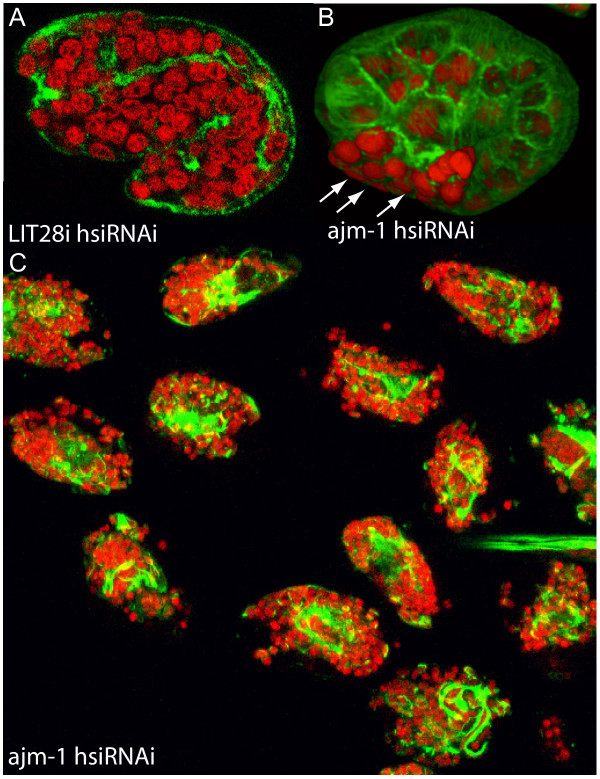
**RNAi against *ajm-1 *in *B. malayi *early embryos**. Stained embryos from control Lit28i hsiRNA-treated (A) or *ajm-1 *hsiRNA-treated (B, C) worms are shown. RNAi control 1.5 fold embryo (A). For comparison, a 1.5 fold embryo displaying morphogenesis defects as a result of loss of AJM-1 (B). The extruded cells (arrows) indicate a compromised junctional integrity of the epidermis. A cluster of late-stage embryos extracted from a uterine fragment, showing robust and consistent phenotypes of cell extrusion and elongation defects (C). Staining with propidium iodide (red) and phalloidin (green) indicate DNA and actin, respectively. A 442 bp fragment of the *B. malayi ajm-1 *cDNA was amplified with primers: ajmF- 5'-TAATACGACTCACTATAGGGGAACGACTATATGTACGGTG -3' and ajmR 5'-TAATACGACTCACTATAGGGGATCATATTGACGACACAGAG -3' where the T7 promoter sequence is underlined and followed by a GG nucleotide (see Methods). An annealing temperature of 60°C was used in the PCR. The cDNA fragment was transcribed and RNA diced to hsiRNA as described. Adult females were soaked for 2 days in 1 μM ajm-1 hsiRNA and tissues fixed on the third day. Control females were kept in the same conditions and included worms cultured without hsiRNA or with a comparable concentration of Lit28i polylinker ShortCut siRNA mix.

We started with γ-tubulin, a cytoskeleton component critical for centrosome-dependent microtubule nucleation and consequently proper spindle formation and cytokinesis [28,29]. A 2 day incubation with 100 nM hsiRNA against *B. malayi *γ-tubulin lead to cytokinesis defects during early embryogenesis, as visualized by our optimized immunostaining procedures (Figure 1), and was sufficient to target all zygotic and very early embryonic divisions. Cytokinesis did not occur in zygotes from γ-tubulin hsiRNA-treated worms resulting in polynucleated 1-cell embryos (Figure 1D, E, F). This suggests that robust phenotypes can be induced by our hsiRNAi procedures at relatively low concentrations of RNA. Similarly, exposure of *B. malayi *to *par-1 *hsiRNA (1 μM) for 2 days resulted in polarity defective embryos. In *C. elegans*, the Ser/Thr kinase PAR-1 is crucial for establishing the embryonic anterior-posterior polarity, and PAR-1 removal leads to defects in posterior spindle rotation and loss of division asynchrony at the 2-cell stage [30]. In *B. malayi *embryos from untreated worms, divisions at the 2-cell stage were asynchronous with the anterior blastomere dividing first and the spindle in the posterior blastomere rotating to align with the anterior-posterior axis (Figure 2A, C) as in *C. elegans*. However, following exposure to *par-1 *hsiRNA, divisions at the 2-cell stage became synchronous and the spindle in the posterior blastomere failed to rotate (Figure 2B, D), again precisely phenocopying defects assigned to removal of the corresponding protein in *C. elegans *[30]. In a third example, that of the epithelial cell junction protein AJM-1, we observed a more severe phenotype in *B. malayi *than in *C. elegans*. Removal of AJM-1 in *C. elegans *results in embryonic elongation defects and developmental arrest [31,32]. After targeting the *B. malayi ajm-1 *transcript by RNAi we not only observed robust elongation defects but also severe ventral closure defects and/or epidermal rupture leading to cell protrusions (Figure 3B, C) entirely consistent with disruption of epithelial junction components in *C. elegans *[32].

The unequivocal phenotypes we report were obtained using hsiRNA concentrations in the range of 0.1 to 1 μM (~1.3 to 13 μg/ml), whereas other studies addressing RNAi in animal parasitic nematodes have generally used much higher long dsRNA concentrations, frequently in the range 1 to 3.5 mg/ml [13,14,20,22,27]. In the few reports on the use of synthetic short interfering RNA (siRNA) or hsiRNA in animal parasitic nematodes, the concentration has typically ranged from 1 to 5 μM, with gene silencing observed in some, but not all, cases [13,22,33]. It appears that the nematode cuticle may be more permeable to smaller RNA molecules [22,33].

We have confirmed phenotypes for all 7 transcripts targeted by this approach including 6 genes expressed during embryogenesis and 1 in adult tissue, comprising cytoskeleton components, some of them regulators or interactors, as well as genes involved in metabolism. This high rate of effective gene targeting contrasts earlier reports of inefficient RNAi-induced silencing in animal parasitic nematodes [13-18]. Our success may be due in part to targeting genes known to show cellular phenotypes in *C. elegans *(6 of 7 cases), although in previous studies efficient silencing in *C. elegans *has not translated well into silencing of orthologs in animal parasitic species. For example of 10 genes that show clear larval phenotypes when subjected to RNAi in *C. elegans*, not one resulted in a phenotype when the orthologous genes in *Haemonchus contortus *orthologs were tested and only two showed any transcript reduction [13]. Our method of RNA delivery may contribute to the effective RNAi results we demonstrate. Most studies have used long dsRNA or one (or a few) synthetic siRNAs that correspond to the gene targeted [14]. Few studies have used hsiRNA mixtures produced enzymatically from longer dsRNA molecules to yield multiple overlapping short RNAs. In one report [22] a variant of the present method was performed using an RNase III digestion protocol which generates predominantly short RNA species (~13 bp) that are not expected to be effective in gene silencing [34]. Unlike other methods employing RNase III, the enzyme conditions used in the present study convert long dsRNA to a mixture of short RNAs ranging from ~18-25 bp, predominantly of around the optimal 21 bp size [35]. Furthermore, since all molecules in the mixture are specific for the targeted gene very potent silencing can be achieved. Conversely, off-target effects are minimized since the molar concentration of any molecule in the mixture that matches a non-target transcript will be very low [35].

## Conclusions

By use of optimized immunostaining protocols we have been able to document cellular phenotypes induced by our newly described method for culturing *B. malayi *in hsiRNA mixtures. Visible cellular defects were observed in all cases tested to date. The entire process can be completed in less than 1 week and is amenable to scale-up, thereby allowing silencing of several genes in parallel. Although RNAi is a proven powerful tool for investigating gene function in many diverse organisms [36], including the model nematode *C. elegans *where it has been used for high-throughput functional genomics [12,37], the technology had previously met with limited success when applied to animal parasitic nematodes [13-18].

The combination of RNAi and immunostaining we report provide critical tools for characterizing developmental processes and unraveling the complex association and transmission pattern of *Wolbachia *endosymbionts in filarial nematodes. More generally, our RNAi methods can be used to investigate components of metabolic pathways in *B. malayi *as well as the function of genes that are either absent or very divergent in the human genome to validate candidate drug targets for subsequent development [38]. Besides our immunofluorescence-based phenotype analysis, RNAi-mediated transcript knockdown can also be confirmed by RT-PCR or monitoring of readily scored phenotypes such as microfilarial output or molting efficiency [21,22]. The general conservation of structure and basic biology of all nematodes raises the exciting prospect that our approach can be deployed with little or no modification to any species amenable to brief *in vitro *maintenance and facilitate a similarly robust reverse genetic approach towards characterization of gene function in some of the worlds most devastating pathogens.

## Competing interests

The authors declare that they have no competing interests.

## Authors' contributions

FL participated in study conception and design, generated RNA, performed the RNAi and immunostaining, and helped draft and edit the manuscript. JF participated in study conception and design, generated RNA, cDNA, and drafted the manuscript. BS participated in study design and helped draft and edit the manuscript. WS participated in study conception and design, and helped draft and edit the manuscript. All authors read and approved the final manuscript.

## Supplementary Material

Additional file 1 **Detailed RNAi and immunostaining protocol**. An enumerated step-by-step protocol with helpful tips. Click here for file

Additional file 2 **Handling *B. malayi *worms and set-up of the culture plate**. Worms are handled under a laminar flow hood using a curved pick and placed in 1 ml WCM (A, B). For multiple hsiRNAi experiments, the 2 worms in each well corresponding to 1 experimental condition can be moved to another plate, in the same corresponding well (C). For a single hsiRNAi experiment over 3 days or less, see Additional file 3. Click here for file

Additional file 3 **Use of a 12-well plate for one hsiRNAi experiment with a matched non-treated control**. At day 1, the plate is divided in two, with the left part used for hsiRNAi, and the right part used for the control worms. The hsiRNA (i.e. 1 μM or ~13.5 μg) is added to the first well. Both treated and control wells receive 1 ml of WCM, then 2 worms as described above. Purple arrows show how the worms are moved every 12 hours to different wells with fresh medium, supplemented with hsiRNA in the case of the treated worms. For larger experiments with several samples, worms are transferred to corresponding wells of new culture plates every 12 hr. Click here for file

Additional file 4 **Preparing worm samples for fixation**. Worms are placed on a glass slide using a curved pick. A razor blade is used to cut the adult worms into smaller fragments (A). This can be monitored under a dissecting microscope. Embryos and adult tissues are transferred by directing the flow of buffer towards a corner of the glass slide and into a microcentrifuge tube (B). Click here for file
