# Supplementary material for: Efficient in vitro RNA interference and immunofluorescence-based phenotype analysis in a human parasitic nematode, Brugia malayi
Source: Parasit Vectors. 2012 Jan 13;5:16. doi: 10.1186/1756-3305-5-16 (PMC3292814; doi:10.1186/1756-3305-5-16)
Supplement: Additional file 1 — Detailed RNAi and immunostaining protocol. An enumerated step-by-step protocol with helpful tips. [file 1756-3305-5-16-S1.DOC]

**Detailed protocol for *in vitro* RNA interference and immunofluorescence-based phenotype analysis in a human parasitic nematode, *Brugia malayi***

**REAGENTS**

Protoscript M-MuLV First Strand cDNA Synthesis Kit (New England Biolabs, Ipswich, MA, USA)

Crimson Taq DNA Polymerase (New England Biolabs)

Gene-specific primers

Note: Include T7 promoter sequence (TAATACGACTCACTATAG) at the primer 5’ ends followed by two guanine bases (GG) to permit transcription of RNA by T7 RNA polymerase and to enhance transcription yield.

HiScribe T7 *In Vitro* Transcription kit (New England Biolabs)

dsRNA Ladder-A (New England Biolabs)

ShortCut RNase III (New England Biolabs)

Paraformaldehyde 32% (Electron Microscopy Sciences, Hatfield, PA, USA)

Propidium iodide 1mg/mL (Molecular Probes, Carlsbad, CA, USA)

Vectashield mounting medium (Vector Laboratories, Burlingame, CA,USA)

Note: Can purchase with DAPI included if wish to visualize DNA and the optional propidium iodide staining steps (Steps 68-70, 83) are not performed

**PROCEDURE**

Note: Use RNase-free plasticware and reagents for Steps 1-48

PREPARATION OF *B. MALAYI* 1^st^ STRAND cDNA

1. Thaw ProtoScript M-MuLV First Strand cDNA Synthesis kit components and store on ice

2. On ice, mix 1 – 6 μl (1 ng – 1μg) RNA with 2 μl Random primer mix

Note: We routinely use 1 μl (~700 ng) *B. malayi* total RNA prepared by established methodology (http://www.filariasiscenter.org/molecular-resources/protocols) as template.

3 Add nuclease-free H_2_O to give 8 μl if necessary

4. Denature at 70^O^C, 5 min, spin briefly, return to ice

5. Add 10 μl M-MuLV Reaction Mix and 2 μl M-MuLV Enzyme Mix

6. Incubate 25^O^C, 5 min then 42^O^C, 1 hr

7. Inactivate enzyme at 80^O^C, 5min

8. Spin briefly then dilute by adding 30 μl H_2_O

9. Determine concentration of aliquot by spectrophotometry

10. Store at -20^O^C

Note: A control reaction using 2 μl H_2_O instead of Enzyme Mix is recommended to detect DNA contamination of the RNA.

PREPARATION OF DNA TEMPLATE FOR *IN VITRO* TRANSCRIPTION

11. To a PCR tube on ice add 15.875 μl H_2_O, 5 μl 5X Crimson Taq Reaction Buffer, 2 μl 2.5 mM dNTP mix, 1 μl of each of 10 μM forward and reverse gene-specific primers, 0.125 μl Crimson Taq polymerase, 1 μl (~100 ng) 1st strand cDNA.

12. Cycle: 95^O^C, 30 sec (1 cycle), 95^O^C, 20 sec; primer dependent annealing temp, 30 sec; 68^O^C, 30 sec (30 to 40 cycles), 68^O^C, 5 min (1 cycle)

13. Confirm desired amplicon by gel electrophoresis of 1 to5 μl aliquot

14. Store remainder at 4^O^C or -20^O^C

Note: The PCR can be scaled up or down and optimized to obtain suitable yield of specific product. A control lacking cDNA template is recommended.

Note: Alternatively, amplicons derived from gDNA templates can be used for *in vitro* transcription if desired.

*IN VITRO* PRODUCTION OF dsRNA

15. Thaw 10X Transcription Buffer and 20X Ribonucleotide Solution Mix from the HiScribe T7 In Vitro Transcription Kit at RT then keep on ice. [Keep 20X High Molecular Weight Mix and T7 RNA polymerase at -20^O^C until use]

Note: If precipitate is visible after thawing, vortex to resuspend.

16. Add 55 μl RNase-free H_2_0, 8 μl 10X transcription Buffer, 4 μl 20X Ribonucleotiode Solution Mix, 5 μl (~1μg) DNA template, 4 μl 20X High Molecular Weight Mix, 4 μl T7 RNA polymerase to give an 80 μl reaction

Note: Between 1 and 2 μg DNA template should be used. The volume of DNA solution used can be balanced by adjusting the volume of H_2_0.

17. Incubate at 42^O^C for 2.5 hr (up to 4 hr is acceptable)

18. Heat RNA to 65^O^C, 1 min, then cool to RT

Note: This step to anneal complementary strands is optional – the transcribed RNA is essentially already all double-stranded.

19. Check 1 μl on a RNase-free agarose gel alongside dsRNA Ladder-A

Note: Electrophoresis at >120V helps minimize RNA degradation

dsRNA PURIFICATION BY ISOPROPANOL PRECIPITATION

20. Add 1 vol RNase-free water

21. Add equal vol isopropanol

22. Add 1/10 vol 3M sodium acetate (pH 5.2)

23. Incubate at RT, 5 min

24. Centrifuge 16,000x g, 10 min at RT

25. Replace supernatant with 1 vol 70% ethanol

26. Incubate at RT, 5 min

27. Centrifuge 16,000x g, 5 min at RT

28. Remove supernatant and air dry pellet

29. Before pellet completely dries resuspend in 50 μl RNase-free H_2_0

PROCESSING OF dsRNA into hsiRNA with ShortCut RNase III

30. To 20 μl RNase-free H_2_0 add 10 μl 10X ShortCut Reaction Buffer, 50 μl dsRNA (the entire prep), 10 μl ShortCut RNase III, 10 μl MnCl_2_

31. Mix and incubate at 37^O^C, 20 min

32. Add 10 μl 10X EDTA to stop reaction

PURIFICATION OF hsiRNA BY ETHANOL PRECIPITATION

33. Add 1/10 vol 3M sodium acetate, pH 5.2, 2 μl RNase-free glycogen (kit) and 3 vols cold 95% ethanol

34. Place at -70^o^C, 30 min (or -20^o^C, 2 hr)

35. Centrifuge 14,000x g, 15 min

36. Replace supernatant with 2 vols 80% ethanol

37. Incubate at RT, 5 min

38. Centrifuge 14,000x g, 5 min

39. Remove supernatant and air dry pellet

40. Resuspend pellet in 60 μl RNase-free H_2_O

41. Check aliquot of hsiRNA on 2% agarose gel alongside siRNA marker

42. Quantify hsiRNA using Nanodrop or a standard spectrophotometer

Note: We obtain yields of 50–60 μg hsiRNA per transcription reaction

*IN VITRO* hsiRNAi INTERFERENCE BY SOAKING

Note: All equipment should be sprayed with 70% ethanol before use under a laminar flow hood

43. Under the hood add hsiRNA to wells of a 12 well cell culture plate

44. Add 1 ml pre-warmed WCM

45. Add 2 worms per well by use of a curved pick (see Additional file 2)

46. Incubate plate with lid at 37^O^C under 5% CO_2_ in air

47. Change medium every 12 hrs by repeating Steps 43-46 (see Additional file 3)

48. Maintain culture for 2 - 5 days (depends on stability of product targeted)

Note: We use a 1μM hsiRNA conc for soaking and increase up to 5 μM if no effect is observed initially. The hsiRNA supply can be preserved by use at 100 nm if robust effects are observed at 1 μM

Note: A control consisting of no hsiRNA or hsiRNA corresponding to a gene with no match to the nematode genome should be included.

Note: 1μM hsiRNA = ~13.5 μg/ml (21 bp x 660 kDa = 13,860). A 2 day exp’t (duplicate wells) with change of medium (1 ml) every 12 hr requires ~108 μg hsiRNA for each pair of treated worms

EMBRYO AND TISSUE COLLECTION AND FIXATION

49. Move the 2 worms from each well to 10 μl 1X PBS on a microscope slide

50. Chop with a razor blade (see Additional file 4, Panel A)

Note: To maximize embryo recovery chop finely; for larger structures cut less then chop the expelled tissues

Note: To specifically facilitate subsequent staining of late stage embryos (after morphogenesis has occurred) proceed to Step 62

51. Wash embryos/tissue to 1.5 ml tube with 180 μl 1x PBS/1% NP-40 (see Additional file 4, Panel B)

52. Add 20 μl 32% paraformaldehyde and 2 vols (~400 μl) heptane

53. Vortex 1 min then place on rotator for 20 min

54. Pellet at ~2000x g (~4600 rpm), 1 min

55. Replace supernatant with 1 ml PBST and rotate for 5 min

To facilitate subsequent staining in embryos up to ~ the 20 cell stage add the following steps:

56. Pellet again and transfer embryos to microscope slide

57. Apply coverslip then downward pressure with paper towel

58. Use tweezers to hold slide in LN_2_ until it stops boiling

59. Remove slide to bench and flick off coverslip with razor

60. Use PBST to wash thawed embryos into 1.5 ml tube (see Additional file 4, Panel B)

61. Proceed to optional RNase step (Step 68) or staining (Step 71)

Note: To facilitate subsequent staining in later stage embryos (after morphogenesis has begun:

62. Wash embryos/tissue to 1.5 ml tube with 180 μl 1x PBS, 20% bleach (see Additional file 4, Panel B)

63. Vortex for no more than 30 sec

64. Add 1 ml PBS, shake and centrifuge immediately ~2000x g (~4600 rpm), 1 min

65. Replace supernatant with 180 μl 1X PBS, 1% NP-40

66. Add 20 μl 32% paraformaldehyde then 2 vols (~400 μl) heptane

67. Follow Steps 53 to 55 then proceed to Step 68 or Step 71

RNase TREATMENT FOR PROPIDIUM IODIDE DNA STAINING (OPTIONAL)

Note: This step should be used when DNA is to be stained with propidium iodide (Step 83) rather than by use of DAPI-containing mounting medium

68. Pellet samples in PBST

69. Replace supernatant with 10 mg/ml RNase in PBST

70. Keep tube on rotator at 4^O^C overnight

Note: For immunofluorescent microscopy or for a confocal equipped with a UV laser the DNA can be stained later by use of DAPI-containing mounting medium

STAINING AND IMMUNOSTAINING

71. Spin samples to remove PBST (or RNase in PBST) supernatant

Note: May store RNase / PBST at 4^O^C short-term for re-use

72. Add 500 μl PBST

73. Add primary antibodies and rotate overnight at 4^O^C

74. Pellet, remove supernatant

Note: The supernatant may be be kept for additional immunostainings

75. Add 1.5 ml fresh PBST to sample and rotate for at least 15 min

76. Pellet and repeat Steps 75 & 76 twice with final resuspension in 500 μl PBST

77. Add secondary fluorochrome-conjugated antibodies at recommended dilution

78. Rotate tube overnight at 4^O^C

79. Pellet samples, discard supernatant

80. Add 1.5 ml PBST and rotate at least 15 min

81. Pellet supernatant, discard supernatant

82. Add 1.5 ml PBST

83. Optional: add 20 μl of 1 mg/ml propidium iodide if not using DAPI-containing mounting medium later

84. Shake tube, 20 sec

85. Pellet again, discard supernatant

86. Add 1.5 ml PBST, shake tube 20 sec

87. Pellet and remove as much supernatant as possible

88. Add ~30 μl mounting medium to tube

Note: May use DAPI-containing mounting medium if propidium iodide not used earlier (Step 83)

89. Mix sample and mounting medium by pipetting up and down

90. Transfer to microscope slide

Note: Larger tissue fragments should be discarded or moved with tweezers to a second slide with > 30 μl mounting medium to prevent floating of the smaller embryos between slide and coverslip

91. Apply coverslips to slides

92. Apply downward pressure with paper towel to embryo slides to blot excess mounting medium and stabilize embryos

93. Seal slides around edges with transparent nail polish

94. Store in dark at 4^O^C

Note: Store slides mounted in DAPI-containing medium for > 1 day to allow tissue penetration

**RECIPES**

Worm culture Medium

RPMI-1640 w/ L-glutamine (Invitrogen, Carlsbad, CA, USA), 1% glucose, 1X antibiotic-antimycotic solution (A-5955; 100X from Sigma-Aldrich, St Louis, MO, USA), 10% Fetal bovine serum -inactivated at 56^o^C for 30 minutes (Invitrogen).

Note: The solution is filtered in a Nalgene filtration bottle (Nalgene, Rochester, NY, USA) in a laminar flow hood. Store at 4^O^C but warm to 37^O^C prior to use.

20X PBS

21.8 g Na_2_HPO_4_, 6.4 g NaH_2_PO_4_, 180 g NaCl, distilled water to 1L. Adjust pH to 7.2 .

Dilute 10-fold in water for use at 1X conc

PBST

Add 2 μl Triton X-100 to each 10 ml 1X PBS

Final conc Triton X-100 is 0.02%

PBST-BSA

Add 20 mg BSA (Fraction V; Fischer Scientific, Pittsburgh, PA, USA) per 1 ml PBST

Final conc BSA is 2%

10 mg/ml RNAseA (Catalog No. R4875, Sigma Aldrich)

Dissolve 10 mg RNase per 1 ml PBST

**TROUBLESHOOTING**

NO PCR PRODUCT (Step 14)

Optimize PCR eg decrease annealing temp, adjust Mg^++^ conc, increase amount of cDNA template

Note: The volume of 1^st^ strand cDNA reaction used as template should not exceed 10% of the PCR reaction.

**EQUIPMENT**

Labquake tube rotator (ThermoScientific, Waltham, MA, USA)

**ACCOMPANYING FIGURES**

Additional file 2. Protocol Figure 1

Handling *B. malayi* worms and set-up of the culture plate.

Worms are handled under a laminar flow hood using a curved pick and placed in 1ml WCM (A,B). For multiple hsiRNAi experiments, the 2 worms in each well corresponding to 1 experimental condition can be moved to another plate, in the same corresponding well (C). For a single hsiRNAi experiment over 3 days or less, see Additional file 3. Protocol Figure 2.

Additional file 3. Protocol Figure 2

Example of use of a 12-well plate for one hsiRNAi experiment with a matched non-treated control. At day 1, the plate is divided in two, with the left part used for hsiRNAi, and the right part used for the control worms. The hsiRNA (i.e. 1µM or ~13.5 µg) is added to the first well. Both treated and control wells receive 1 ml of WCM, then 2 worms as described above. Purple arrows show how the worms are moved every 12 hours to different wells with fresh medium, supplemented with hsiRNA in the case of the treated worms. For larger experiments with several samples, worms are transferred to corresponding wells of new culture plates every 12 hr.

Additional file 4. Protocol Figure 3

Preparing the samples for fixation

Worms are placed on a glass slide using a curved pick. A razor blade is used to cut the adult worms into smaller fragments (A). This can be monitored under a dissecting microscope. Embryos and adult tissues are transferred by directing the flow of buffer towards a corner of the glass slide and into a microcentrifuge tube (B).
