# Supplementary figures and images for: Efficient in vitro RNA interference and immunofluorescence-based phenotype analysis in a human parasitic nematode, Brugia malayi
Source: Parasit Vectors. 2012 Jan 13;5:16. doi: 10.1186/1756-3305-5-16 (PMC3292814; doi:10.1186/1756-3305-5-16)

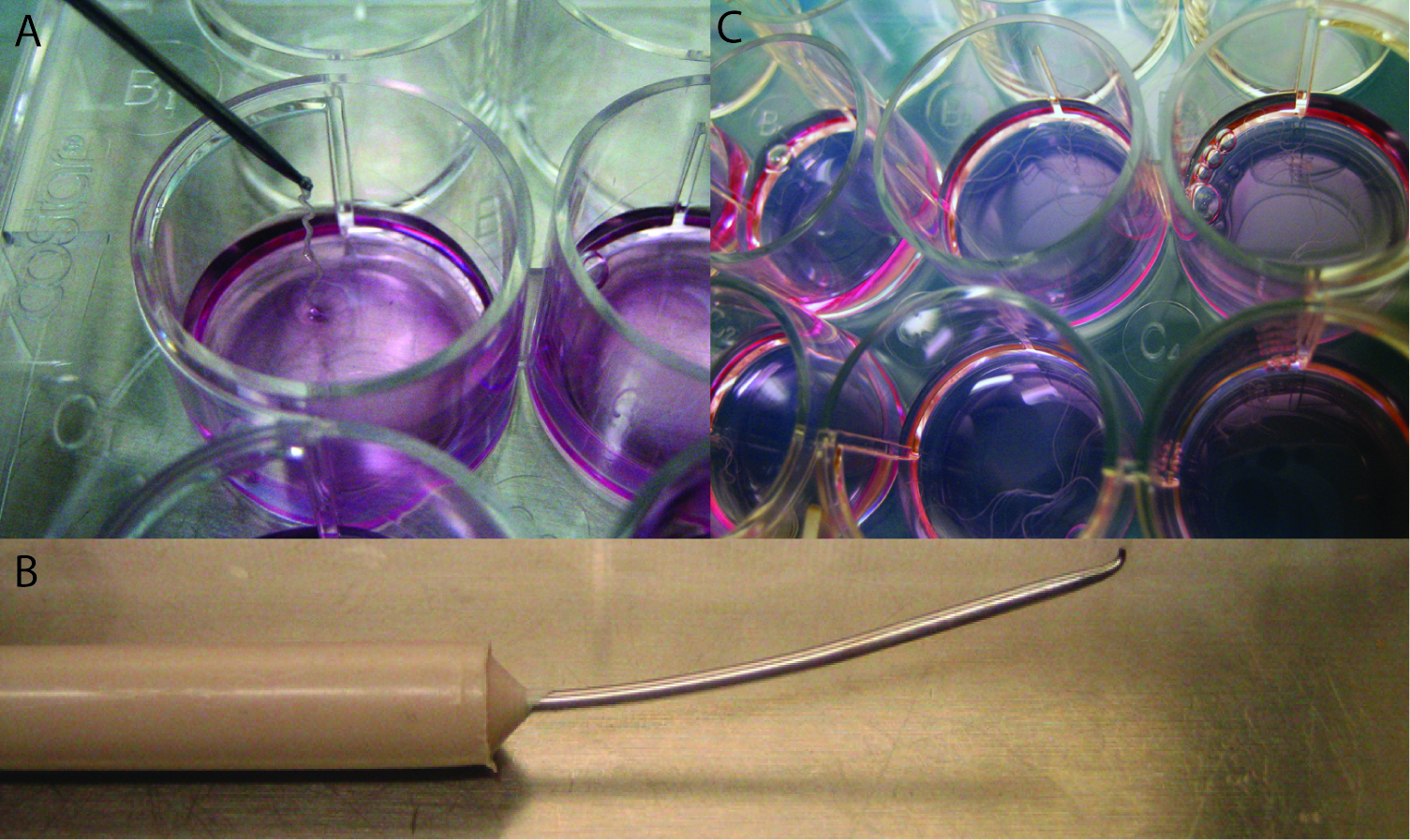

Supplement: Additional file 2 — Handling B. malayi worms and set-up of the culture plate. Worms are handled under a laminar flow hood using a curved pick and placed in 1 ml WCM (A, B). For multiple hsiRNAi experiments, the 2 worms in each well corresponding to 1 experimental condition can be moved to another plate, in the same corresponding well (C). For a single hsiRNAi experiment over 3 days or less, see Additional file 3. [file 1756-3305-5-16-S2.TIFF]

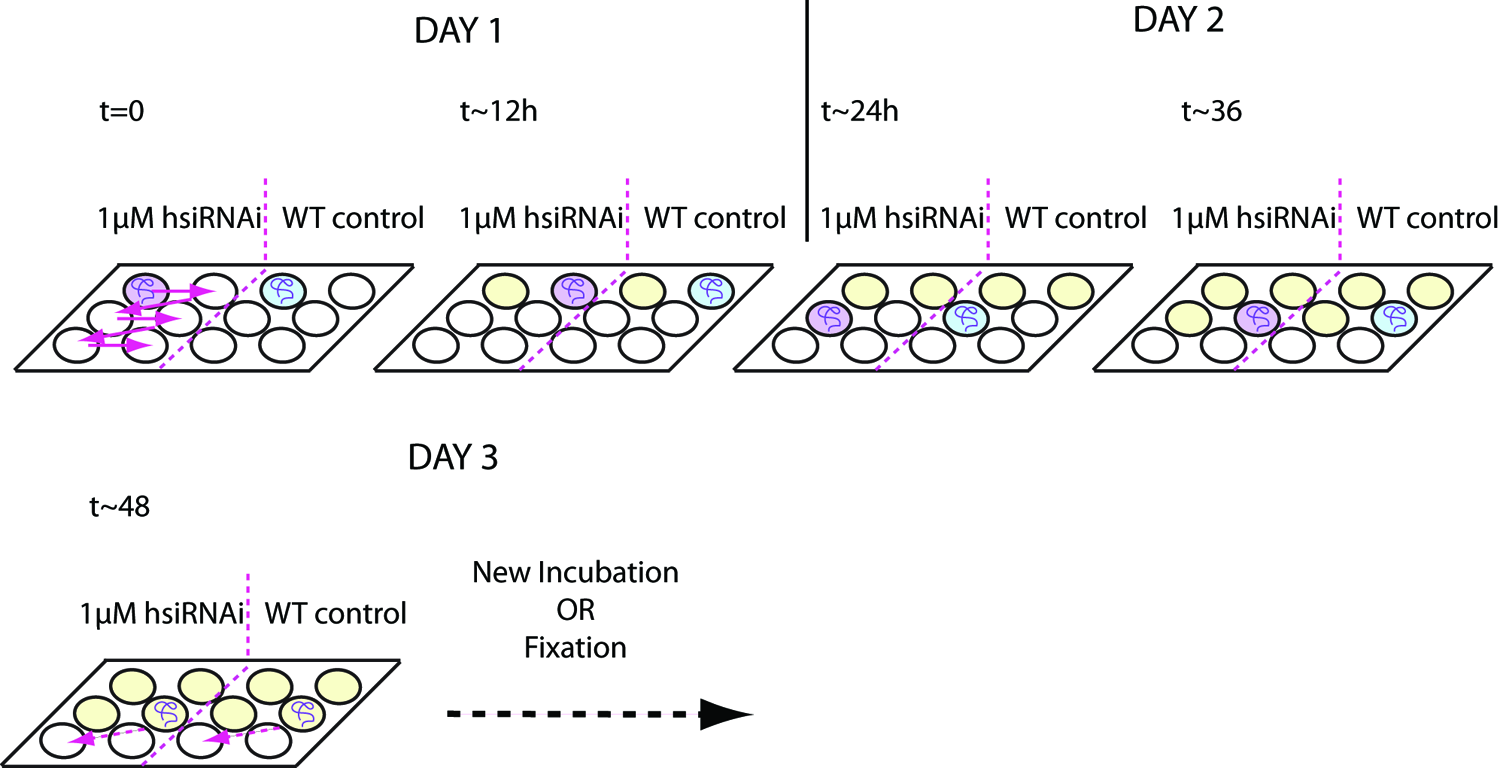

Supplement: Additional file 3 — Use of a 12-well plate for one hsiRNAi experiment with a matched non-treated control. At day 1, the plate is divided in two, with the left part used for hsiRNAi, and the right part used for the control worms. The hsiRNA (i.e. 1 μM or ~13.5 μg) is added to the first well. Both treated and control wells receive 1 ml of WCM, then 2 worms as described above. Purple arrows show how the worms are moved every 12 hours to different wells with fresh medium, supplemented with hsiRNA in the case of the treated worms. For larger experiments with several samples, worms are transferred to corresponding wells of new culture plates every 12 hr. [file 1756-3305-5-16-S3.TIFF]

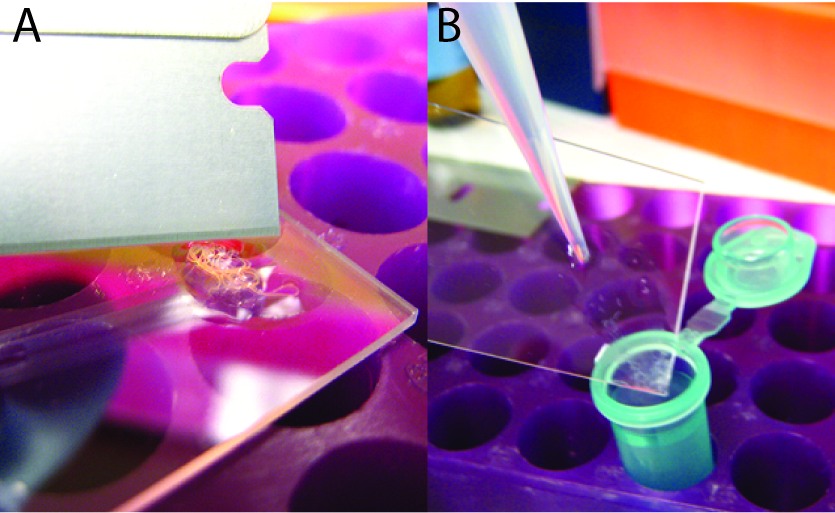

Supplement: Additional file 4 — Preparing worm samples for fixation. Worms are placed on a glass slide using a curved pick. A razor blade is used to cut the adult worms into smaller fragments (A). This can be monitored under a dissecting microscope. Embryos and adult tissues are transferred by directing the flow of buffer towards a corner of the glass slide and into a microcentrifuge tube (B). [file 1756-3305-5-16-S4.TIFF]
